# Adagrasib in Advanced Lung Adenocarcinoma: Therapeutic Benefit With Acute Risk Considerations

**DOI:** 10.51894/001c.166007

**Published:** 2026-07-31

**Authors:** Tumodir Abdallah, Ahmed Chaudhary, Khalil Kamal

**Affiliations:** 1 DMC - Sinai Grace

## 119

### Introduction

Lung adenocarcinoma is the most common subtype of primary lung cancer, accounting for approximately half of all cases, and remains the leading cause of cancer related mortality worldwide. Recent therapeutic innovations in targeted therapies have significantly changed disease management. Adagrasib, a selective inhibitor of the KRAS G12C mutation, is approved for the treatment of adults with KRAS G12C–mutated, locally advanced or metastatic non–small cell lung adenocarcinoma who have received at least one prior systemic therapy. Its adverse effect profile most commonly includes gastrointestinal toxicities and hepatotoxicity. This case highlights a rare and poorly documented cardiovascular complication observed following initiation of adagrasib therapy.

### Case Description

A 63-year-old woman with stage IV lung adenocarcinoma with brain metastases and a chronic right-sided pleural effusion presented with worsening dyspnea a week after starting adagrasib. She reported increased gastrointestinal symptoms and pleural catheter output. Baseline oxygen requirement was 2 L nasal cannula; on presentation she required 15 L via non-rebreather mask and was intubated for hypoxic respiratory failure. Imaging revealed a new large pericardial effusion. Echocardiogram demonstrated a circumferential effusion measuring up to 3.5 cm. She had no prior history of pericardial disease. Pericardiocentesis drained 1.2 L of bloody fluid. Pericardial fluid analysis showed. Fluid cytology demonstrated bloody, red fluid with 1, 343, 470 RBC and 1591 nucleated cells. LDH and glucose were not obtained. She was extubated, chest tubes were removed, and she was discharged on 3 L nasal cannula. After oncology consultation, adagrasib was resumed with outpatient follow-up,

### Discussion

The etiology of this patient’s pericardial effusion was uncertain, with both malignant involvement and adagrasib-associated toxicity considered. Malignant pericardial effusion is a complication of advanced lung adenocarcinoma; however, pericardial effusion has also been reported as a rare adverse event associated with KRAS G12C inhibitors, often occurring early after treatment initiation. Distinguishing between malignant and treatment-related effusions remains challenging and requires comprehensive evaluation. This case highlights the importance of maintaining a broad differential diagnosis when evaluating pericardial effusion in patients receiving KRAS G12C inhibitors. Increased awareness and collaboration between oncology and cardiology teams are essential to balance effective cancer therapy with potential cardiovascular risks.

